# Plain Packaging in Tobacco Control: A Bibliometric Analysis

**DOI:** 10.7759/cureus.40143

**Published:** 2023-06-08

**Authors:** Vedha VPK, Amit V Mahuli, Shahwar Kazmi

**Affiliations:** 1 Public Health Dentistry, Rajendra Institute of Medical Sciences, Ranchi, IND; 2 Public Health Dentistry and Preventive Dentistry, Dental College, Rajendra Institute of Medical Sciences, Ranchi, IND; 3 School of Health and Related Research, University of Sheffield, Sheffield, GBR

**Keywords:** warning labels, tobacco, standardized packaging, plain packaging, fctc

## Abstract

The tobacco epidemic is affecting 8 million people with 1.2 million deaths worldwide. The Framework Convention on Tobacco Control (FCTC) was adopted by the World Health Organization (WHO) Member States in 2003 to counter the growing tobacco menace. Articles 11 and 13 of WHO FCTC suggest plain packaging of tobacco products to reduce the visibility and make tobacco products look less attractive. The current bibliometric analysis was conducted to analyze the visibility and impact of the scientific productions contributing to plain packaging globally. The bibliometric analysis allowed a quantitative analysis of all scientific publications indexed in Scopus. The sample was defined using the keywords “plain packaging OR standardized packaging” AND “tobacco.” Five broad bibliometric domains were assessed for analysis: namely, scientific production, authors, sources or journals, countries, and thematic areas using R programming v4.2.2 and VOSviewer. The total number of documents published regarding plain packaging in tobacco control from 1992 till mid-2022 was determined. Australia tops the list with 99 publications, followed by the United Kingdom, United States, New Zealand, Canada, France, India, Netherlands, Spain, and Egypt. The author citation network showcases the link between the 21 top documents, with a minimum of 50 citations per document. The two main indicators assessed were the total number of articles published in the journal and the h index. Bibliometric indicators in this study illustrated that scientific publications/efforts to implement the WHO FCTC guideline concerning plain packing laws were neglected in most countries.

## Introduction and background

The tobacco epidemic is a chronic public health threat that kills more than 8 million people yearly, including the 1.2 million fatalities attributed to second-hand smoking. There is no safe level of exposure to any tobacco product, regardless of the type [1]. More than 80% of the world’s 1.3 billion tobacco users live in low- and middle-income countries and account for most of the mortality and morbidity caused by tobacco use [1]. The economic impact of tobacco use is very significant, including the healthcare costs for tobacco-attributable diseases and lost capital from tobacco-attributed diseases.

The Framework Convention on Tobacco Control (FCTC) was adopted by the World Health Organization (WHO) Member States in 2003 with 182 parties to counter the growing tobacco menace. It outlines a wide range of evidence-based methods with 38 articles for combating the tobacco epidemic. Articles 6 through 14 are concerned with strategies for reducing tobacco demand, and, specifically, Articles 11 and 13 urge parties to adopt plain packaging. Article 11 states, “Parties should consider adopting measures to restrict or prohibit the use of logos, colours, brand images or promotional information on packaging other than brand names and product names displayed in a standard colour and font style (plain packaging).” Article 13 states, “Packaging, individual cigarettes or other tobacco products should carry no advertising or promotion, including design features that make products attractive” [2].

A total of 127 countries have implemented health warning labels covering at least 30% of the principal display areas of tobacco packages, including 19 countries having labels covering more than 75% of the principal display of tobacco packages [3]. Few countries have taken the implementation of articles one step further by implementing plain packaging.

Plain packaging or standardized tobacco packaging forecloses the display of brands, logos, or other promotional materials outside, inside, or connected to the packaging as well as on individual products. It also entails that the packaging has a uniform plain color and texture. Only the brand name, product name, quantity, and contact information may be printed on the packaging, along with other essential details, such as health warnings and tax stamps, in a standard typeface. This way, the visual appeal and attractiveness of the tobacco packages reduce significantly and the impact of health warning labels is also increased by decreasing the capacity of tobacco packaging to mislead consumers regarding the risks and negative effects of tobacco [4].

Australia was the first country in the world to implement plain packaging legislation, followed by several other countries. To date, 18 countries have passed similar laws enacting plain packaging [4]. Further, many countries are either on the verge of adopting plain packaging or considering it seriously. Literature suggests that plain packaging, implemented in tandem with larger and new pictorial health warning labels, reduces the pack’s appeal and increases the noticeability and effectiveness of the health warnings, thereby impacting the attitudes of tobacco users [5].

Plain packaging would be an effective tool in supplementing the global efforts toward demand reduction strategy by addressing Article 11 and Article 13 of FCTC, as mentioned earlier, along with Article 12: education, communication, training, and public awareness using health warning labels. With only 10% of the signatories of FCTC having implemented plain packaging, the current bibliometric analysis was conducted with the objective of analyzing the visibility and impact of the scientific productions contributing to the area of interest thus far [4].

## Review

Methodology

Bibliometric analysis is secondary research without any human subject involvement. Ethical approval was not required for this study. The bibliometric analysis allowed a quantitative analysis of all scientific publications indexed in Scopus. Scopus was primely used as it is a peer-reviewed database that includes many articles from more than 22,000 publishers [6]. Furthermore, the Scopus database has been used in several bibliometric analyses as it combines the attributes of both Web of Science and PubMed. The sample was defined using the keywords: “plain packaging OR standardized packaging” AND “tobacco.” Article titles, abstracts, and keywords were searched using the keywords. The search was restricted to articles published in the English language but was inclusive of all articles published to date. Five broad bibliometric domains were assessed for analysis, namely, scientific production, authors, sources or journals, countries, and thematic areas.

The parameters of bibliometric analysis including citation and bibliographical information including authors, citation count, affiliations, keywords, journals, and impact factor were exported in the BibTeX format and transferred to R software for further analysis. The collected data were used to analyze the visibility of the topic of plain packaging in tobacco through the annual citations per document and per year, author appearances, the impact of the topic through the annual growth rate, international co-authorships percentage, annual scientific production, scientific productivity, and factorial map of the most cited documents. Only the 10 top-ranked measures of each parameter were taken into consideration. The 10 topmost productive countries were also checked for their implementation status of plain packaging in tobacco products. Additionally, bibliometric networks were created using VOSviewer software. Journal rankings, ISSN number, and h index were obtained from Journal Citation Reports (JCR) and Scimago Journal and Country Rank (SJR).

Results

Production

**Figure 1:**
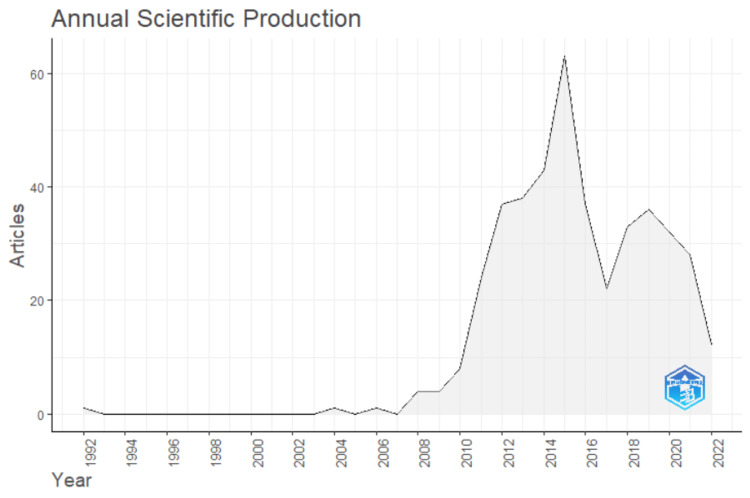
Annual scientific production of plain packaging in tobacco (source: Bibliometrix-R tool).

The total number of documents published regarding plain packaging in tobacco control from 1992 till mid-2022 was found to be 424, including 284 articles, 37 reviews, 13 editorials, and 90 other documents with an average of 15 documents per year. In total, there were 908 authors involved in the publications, with 87 having single-authored documents. The documents per author ratio was found to be 0.467, with 118 single-authored documents and 22.88% international co-authorships (Table 1). Figure 1 depicts the three-decade annual scientific production with a growth rate of 15.74%. Beginning with one publication in 1992, the curve surges in 2011 and hits a peak in 2015. As only the articles published until June 2022 were considered, the curve seems to decline in the current year.

**Table 1:** Main information of plain packaging in tobacco bibliometric analysis (source: Bibliometrix-R tool).

	Description	Results
Main information about the data	Timespan	1992–2022
	Sources (journals, books, etc.)	171
	Documents	424
	Annual growth rate %	15.74
	Document average age	6.35
	Average citations per document	12.86
	Average citations per year per document	1.554
	References	13,920
Document types	Articles	284
	Reviews	37
	Editorials	13
	Other	90
Document contents	Keywords plus (ID)	1,069
	Author’s keywords (DE)	522
Authors	Authors	908
	Author appearances	1,472
	Authors of single-authored documents	87
Authors collaboration	Single-authored documents	118
	Documents per author	0.467
	Co-authors per document	3.47
	International co-authorships %	22.88

Authors

**Figure 2:**
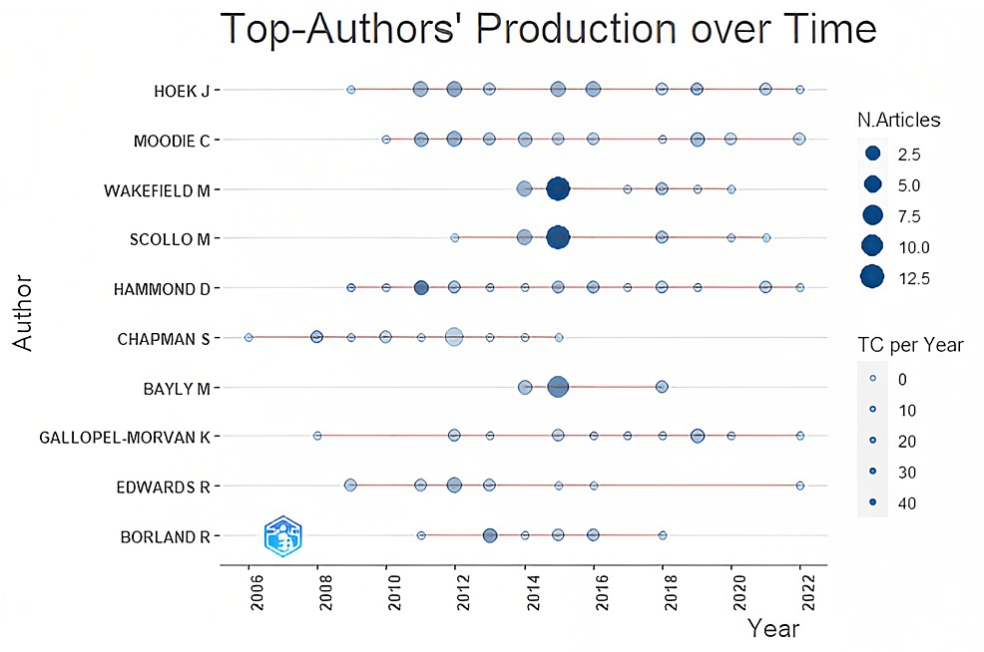
Top authors’ production over time (source: Bibliometrix-R tool).

The bibliometric study assisted us in drawing the following findings about the authors: authors of the most cited documents, authors’ production over time, co-citation network, and authors’ collaboration network. Table 2 lists the authors of the document that received the most citations worldwide in plain packaging. Figure 2 depicts the authors’ production over time along with the total number of documents and total citations per year. The circle sizes represent the number of documents published, while the shades represent the total citations (TC) count, with darker shades representing more citations.

**Table 2:** Most relevant authors and highly cited documents globally (source: Bibliometrix-R tool).

Documents	Total citations	Total citations per year	Normalized total citations
Bansal-Travers et al. 2011, Am J Prev Med [7]	167	113.92	4.77
Freeman et al. 2008, Addiction [8]	137	9.13	1.92
Germain et al. 2010, J Adolesc Health [9]	134	10.31	4.58
Wakefield et al. 2008, Tob Control [10]	123	8.20	1.73
Hammond et al. 2009, Eur J Public Health [11]	122	8.71	2.22
Wakefield et al. 2013, Bmj Open [12]	103	10.30	5.64
Munafò et al. 2011, Addiction [13]	96	8.00	2.74
Thrasher et al. 2011, Health Policy [14]	86	7.17	2.45
Wakefield et al. 2015, Tob Control [5]	83	10.38	5.96
Moodie et al. 2011, Tob Control [15]	80	6.67	2.28

**Figure 3:**
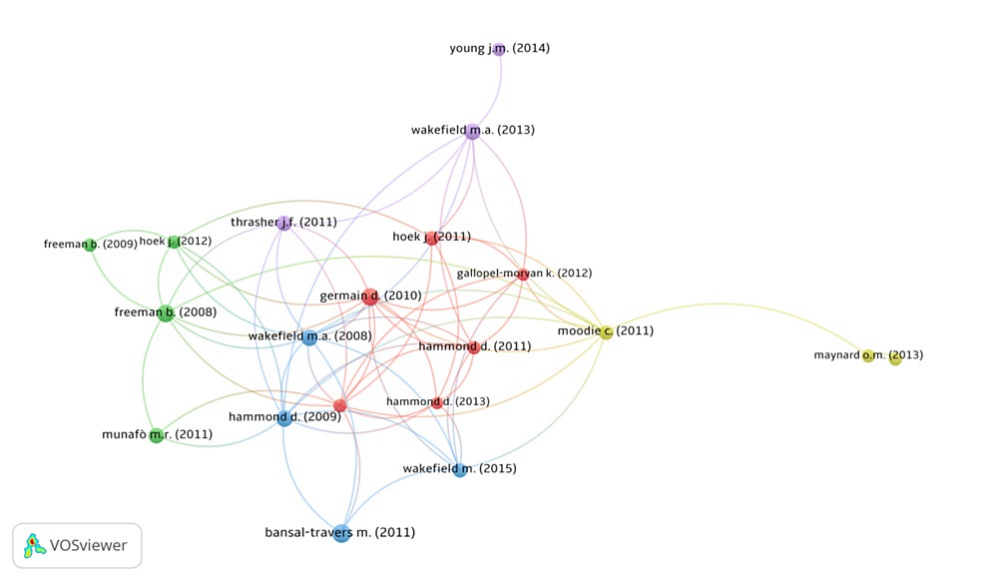
Citation network map (minimum number of citations per document set at 50: 21 articles were found) (source: VOSviewer software).

**Figure 4:**
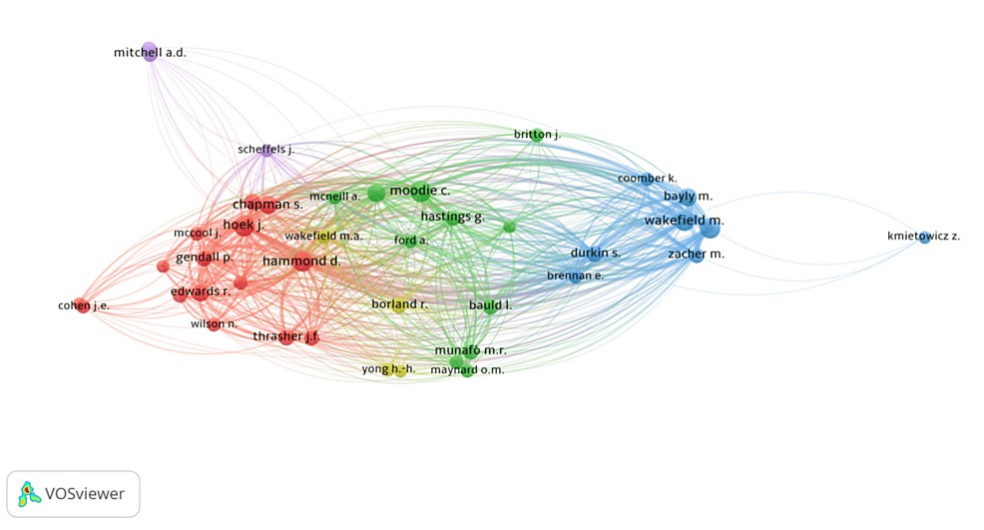
Top authors’ network map (authors were required to have at least five publications for inclusion: 41 met the criteria) (source: VOSviewer software).

**Figure 5:**
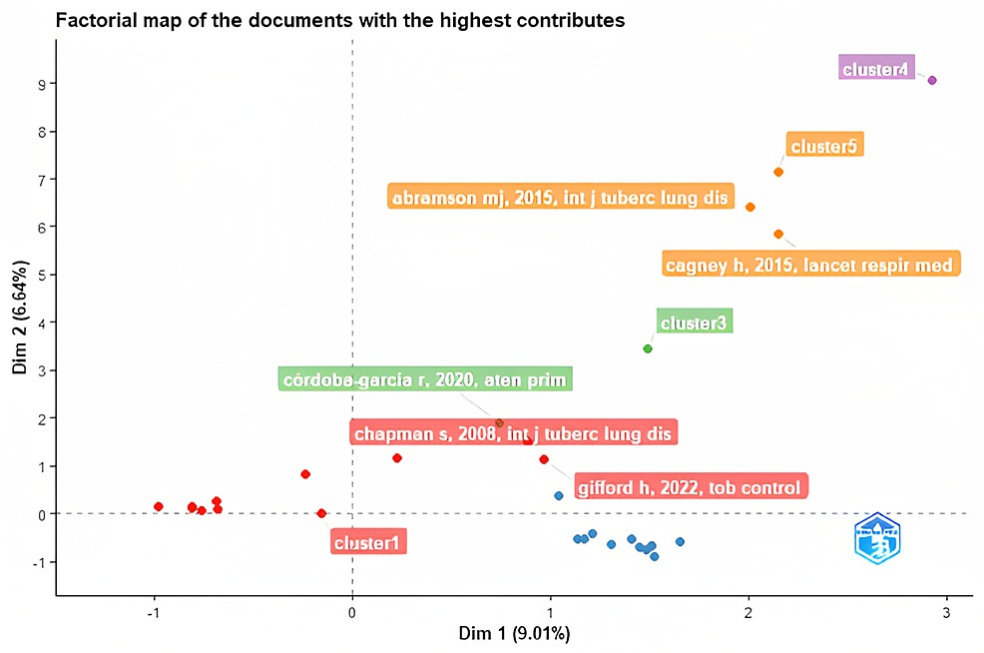
Factorial map of the documents with the highest contributions (source: Bibliometrix-R tool).

Figure 3 and Figure 4 demonstrate the author citation network and the author contribution network, respectively. The author citation network showcases the link between the 21 top documents, which has a minimum of 50 citations per document. The various circle sizes denote the number of citations received per document. The author contribution network depicts the link between the top authors, and only those with a minimum of five publications were included. Figure 5 depicts the factorial map emphasizing the documents that contributed the most. The Bibliometrix-R tool enables factorial analysis to evaluate the conceptual structure of the bibliometric data based on data closeness using the correspondence analysis multivariate statistical technique. Keywords, the number of documents per author, and TC are the dimensions or parameters that are considered. The documents are separated into five clusters across two dimensions or factors, as shown in the figure. As they lie under the positive quadrants of both dimensions, two documents from cluster 1, two documents from cluster 3, one document from cluster 4, and three documents from cluster 5 are ranked with the highest contributions.

Journals

**Figure 6:**
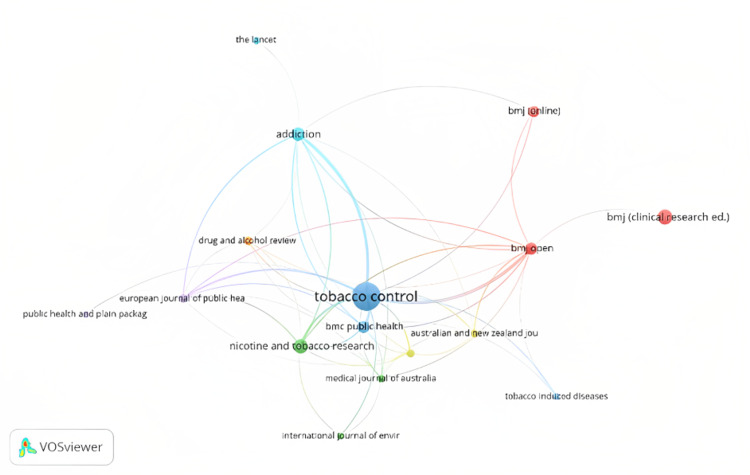
Top sources network map (minimum number of documents of a source set as five: 16 journals met the criteria) (source: VOSviewer software).

Regarding the sources or journal-level bibliometric analysis, the two main indicators assessed were the total number of articles published in the journal and the h index. Elaborating the concept of the h index: an author having h number of articles published in the journal and having h or more citations, therefore, the article’s performance directly impacts the journal’s performance. In other words, the higher the h-index, the better the journal. The journal performance and impact metrics are listed in Table 3. Figure 6 portrays the network map of the top journals which had a minimum of five documents contributed from their source.

**Table 3:** Journal performance metrics of the top 10 sources (source: Bibliometrix-R tool and authors’ compilation).

Journals	Articles	Citations	ISSN	H index
Tobacco Control	69	1,520	0964-4563	129
BMJ	30	50	1756-1833	453
Nicotine and Tobacco Research	18	305	1462-2203	119
Addiction	16	465	0965-2140	202
BMC Public Health	12	217	1471-2458	159
BMJ Open	12	289	2044-6055	121
Drug and Alcohol Review	7	54	0959-5236	81
European Journal of Public Health	7	228	1101-1262	99
Australian & New Zealand Journal of Public Health	6	34	1326-0200	80
Medical Journal of Australia	6	103	0025-729X	137

Countries

**Figure 7:**
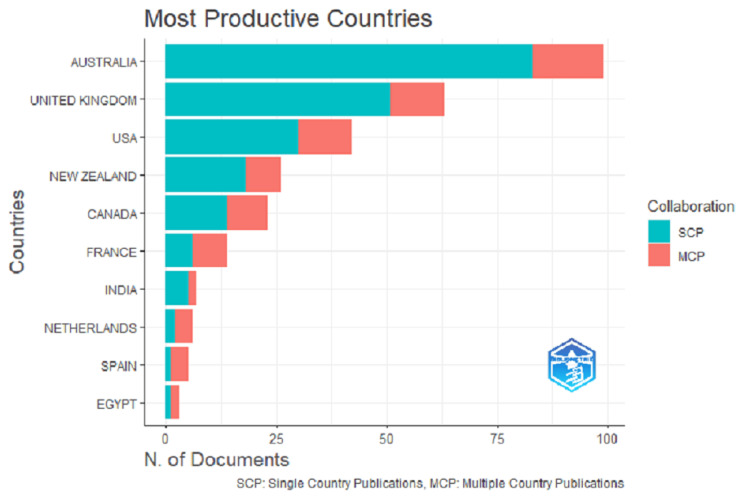
Most productive countries (source: Bibliometrix-R tool).

**Figure 8:**
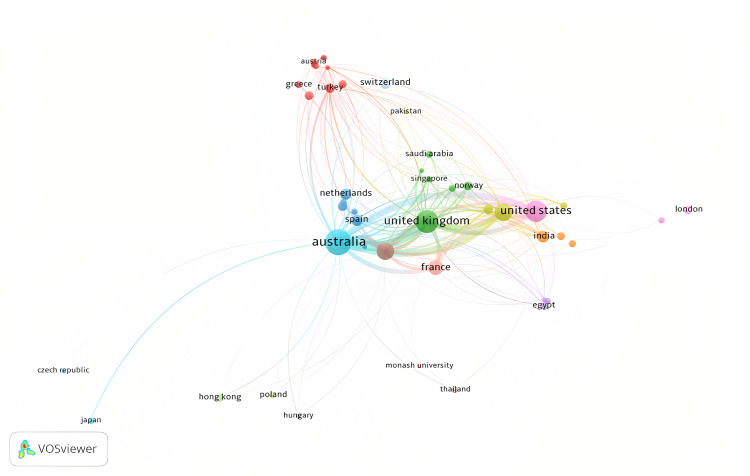
Country collaboration map: network map of all countries with publications on plain packaging in tobacco control (source: VOSviewer software).

**Figure 9:**
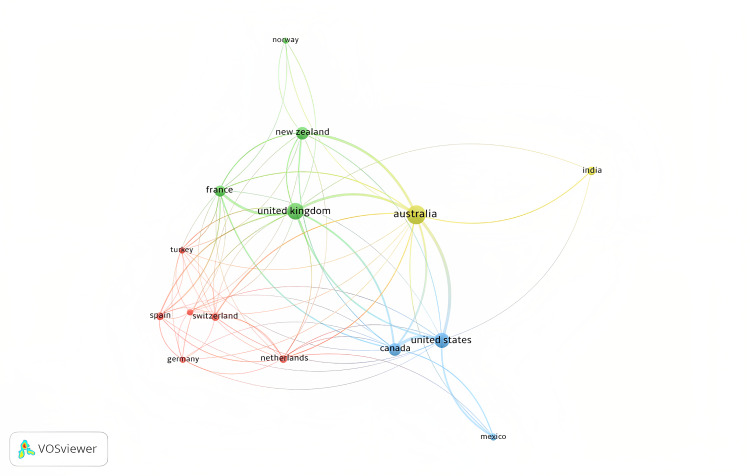
Most contributing affiliations: co-authorship country network map (minimum number of documents of a country set as five) (source: VOSviewer software).

While analyzing the country with the most contributions, Australia tops the list with 99 publications, followed by the United Kingdom, United States, New Zealand, Canada, France, India, Netherlands, Spain, and Egypt (Figure 7). With respect to the top 10 countries with the maximum number of citations, the list was similar to the countries with the most contributions, except for Hong Kong, Norway, and Nepal replaced India, Netherlands, and Spain (Table 4). A network map of all the countries with publications on plain packaging in tobacco control is shown in Figure 8, while the global-level author collaboration network linkage is shown in Figure 9.

**Table 4:** Top 10 countries with the maximum number of citations.

Country	Total citations	Average article citations
Australia	1,983	20.030
United Kingdom	999	15.857
United States	679	16.167
Canada	518	22.522
New Zealand	453	17.423
France	141	10.071
Hong Kong	80	26.667
Egypt	29	9.667
Norway	28	9.333
Nepal	27	27.000

Thematic Analysis

**Figure 10:**
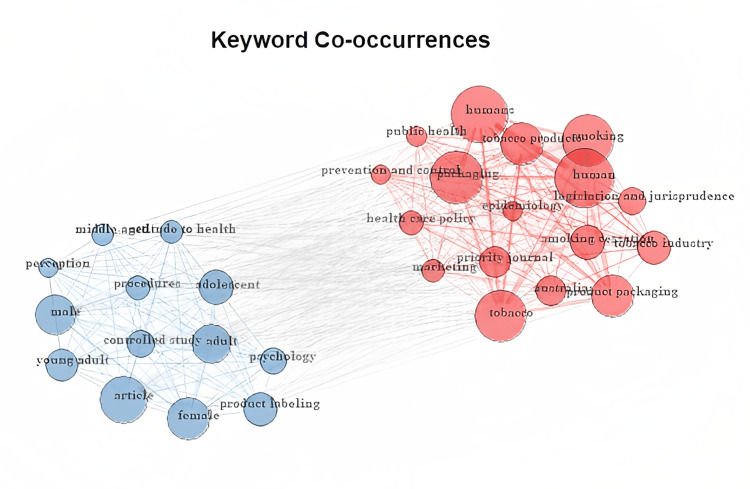
Keyword co-occurrences (source: Bibliometrix-R tool).

**Figure 11:**
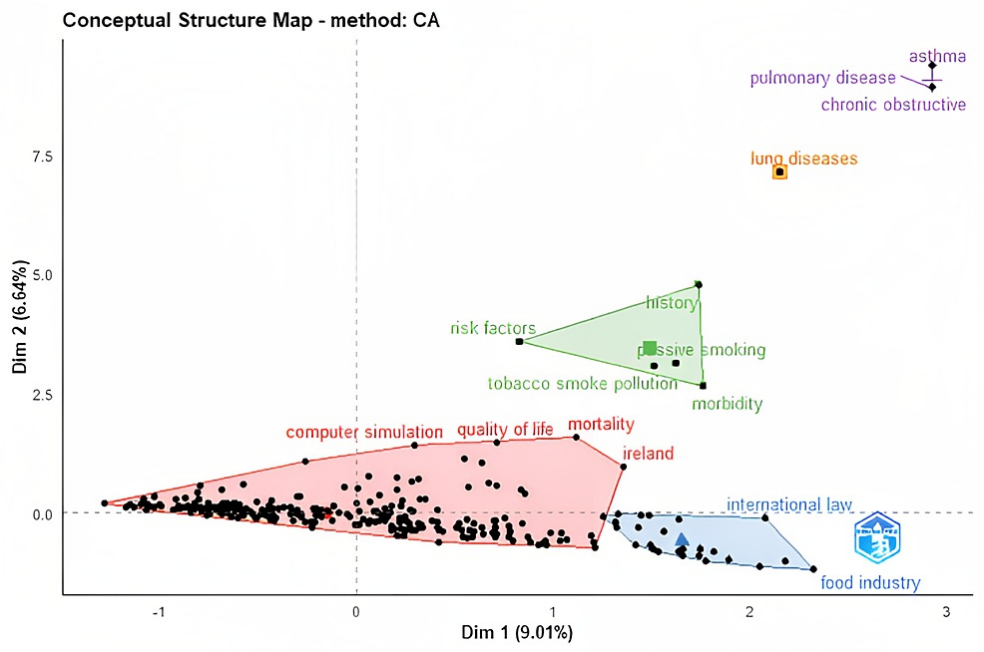
Conceptual structure map (source: Bibliometrix-R tool).

The Bibliometrix-R tool permits highlighting the keyword co-occurrences used in the plain packaging studies and tracking the evolution of the research field over time using the trend topic function (Figure 10). The conceptual structure map shown in Figure 11 gives a high-level overview of the keyword clusters examined from the bibliometric data by concentrating on the keywords and establishing their similarity between the study areas. By identifying their commonalities, five clusters were established using the multi-correspondence analysis multivariate statistical technique.

Discussion

Implementing plain packaging requires understanding how different nations have advanced in plain packaging scientific research. Such an understanding is vital for the development of an effective plan to implement the law with strong political will. The aim of this bibliometric analysis was to statistically evaluate the published literature regarding plain packaging and measure the visibility and influence of publications in the scientific community. The scientific production graph generated by Bibliometrix-R corresponded with the implementation pattern of plain packaging. The curve commenced with a letter published in the World Health Forum in 1992 and started to rise in 2011 when Australia enacted the plain packaging legislation. There was a peak in 2015 around when almost eight countries implemented the legislation [4]. The study of scientific productivity further reported a 15.74% yearly increase rate in research output, with over 13,920 references cited across 424 documents in 171 sources with 908 authors being involved. The annual citations per document were found to be 12.86, thereby reinforcing the need for active participation in this area globally.

Based on the progress and the extent of implementation of plain packaging, it is evident how scientific research related to the field of interest has impacted the scientific community. In line with Articles 11 and 13, though highly commendable efforts have been made to display pictorial and textual health warning labels, with respect to plain packaging, only 10% of the signatories to WHO FCTC have implemented plain packaging. Additionally, among the 18 countries which have implemented only six, namely, Australia, New Zealand, Ireland, Canada, Saudi Arabia, and Singapore, cover all tobacco product packaging [4]. Even if the rest of the world moves toward enacting plain packaging laws, it may be highly challenging to do so because of the litigations, lobbying, public relations media campaigns, threatening the closure of manufacturing facilities, claiming increasing illicit trade, increased theft from vendors, and third-party front groups triggered by the tobacco industries [16].

The country-level analysis revealed Australia as the most active and most productive country with a total of 99 publications (83 single country publications + 16 multiple country publications) and has received the maximum number of total citations (1,983). While reviewing the top 10 list of most productive countries, it was interesting to learn that four out of the 10 most productive countries have not yet implemented plain packaging legislation (United States, Hong Kong, Egypt, and Nepal). Similar results were obtained from the top-ranked nations with the maximum number of citations (United States, India, Spain, and Egypt). This clearly suggests that the topic has been a great area of interest and that countries are making every effort beyond pictorial and textual health warning labels to put plain packaging laws into effect that will make tobacco products less appealing. The result at the same time depicts that only six (Australia, United Kingdom, New Zealand, Canada, France, and the Netherlands) out of 18 countries that have implemented plain packaging are most active in terms of publications. The results of the current study urge the remaining countries to publish more data on the experiences, challenges faced, and steps taken to tackle the giant tobacco industry’s interference.

To our knowledge, this bibliometric study is the first to comprehensively estimate the literature on plain packaging at a global level. This study provides researchers with a global overview, who wish to learn more and contribute to the field of plain packaging. The current bibliometric analysis has the drawback of taking into consideration only articles published in one database for bibliometrics.

## Conclusions

The study highlights the gaps in the policy implementation of plain packaging guidelines per the WHO FCTC in different countries around the world. Bibliometric indicators in the current study illustrated that scientific publications/efforts to implement the WHO FCTC guidelines about plain packaging laws were neglected in most countries, and further research output in this domain should focus on the best practices highlighting specifics of implementation along with the steps taken to overcome the interference of tobacco industry along with the effectiveness of plain packaging in tobacco control.
